# Author Correction: The genetic legacy of continental scale admixture in Indian Austroasiatic speakers

**DOI:** 10.1038/s41598-019-42195-w

**Published:** 2019-04-10

**Authors:** Kai Tätte, Luca Pagani, Ajai K. Pathak, Sulev Kõks, Binh Ho Duy, Xuan Dung Ho, Gazi Nurun Nahar Sultana, Mohd Istiaq Sharif, Md Asaduzzaman, Doron M. Behar, Yarin Hadid, Richard Villems, Gyaneshwer Chaubey, Toomas Kivisild, Mait Metspalu

**Affiliations:** 1 Department of Evolutionary Biology, Institute of Cell and Molecular Biology, University of Tartu, Tartu, 51010 Estonia; 2 Estonian Biocentre, Institute of Genomics, University of Tartu, Tartu, 51010 Estonia; 3 APE Lab, Department of Biology, University of Padova, Padova, 35121 Italy; 4 Centre for Comparative Genomics, Murdoch University, Murdoch, 6150 Australia; 5 The Perron Institute for Neurological and Translational Science, Sarich Neuroscience Research Institute, Nedlands, 6009 Australia; 6 Department of Orthopedic and Traumatology, Hue University of Medicine and Pharmacy, Hue University, 06 Ngo Quyen street, Vinh Ninh ward, Hue, Vietnam; 7 Department of Oncology, Hue University of Medicine and Pharmacy, Hue University, 06 Ngo Quyen street, Vinh Ninh ward, Hue, Vietnam; 8 Centre for Advanced Research in Sciences (CARS), DNA Sequencing Research Laboratory, University of Dhaka, Dhaka, 1000 Bangladesh; 9 The Genomic Laboratory, The Simon Winter Institute for Human Genetics, The Bnai-Zion Medical Center, 7 Golomb St., Haifa, 31048 Israel; 10 Department of Human Genetics, Katholieke Universiteit Leuven, Leuven, 3000 Belgium; 11 Cytogenetics laboratory, Department of Zoology, Banaras Hindu University, Varanasi, 221005 India

Correction to: *Scientific Reports* 10.1038/s41598-019-40399-8, published online 07 March 2019

This Article contains errors in the Methods section, under subsection ‘Samples collection and genotyping’.

“All genotyped data will be made publicly available on the ebc.ee/free_data website and NCBI-GEO public repository (accession number XXXX)”.

should read:

“All genotyped data will be made publicly available on the ebc.ee/free_data website and NCBI-GEO public repository (accession number GSE126882)”.

