# Supplementary material for: The genetic legacy of continental scale admixture in Indian Austroasiatic speakers
Source: Sci Rep. 2019 Mar 7;9:3818. doi: 10.1038/s41598-019-40399-8 (PMC6405872; doi:10.1038/s41598-019-40399-8)
Supplement: Supplementary file 1 — Supplementary Figures and Tables [file 41598_2019_40399_MOESM1_ESM.docx]

**The genetic legacy of continental scale admixture in Indian Austroasiatic speakers**

Kai Tätte, Luca Pagani, Ajai K. Pathak, Sulev Kõks, Binh Ho Duy, Xuan Dung Ho, Gazi Nurun Nahar Sultana, Mohd Istiaq Sharif, Md Asaduzzaman, Doron M. Behar, Yarin Hadid, Richard Villems, Gyaneshwer Chaubey, Toomas Kivisild, Mait Metspalu


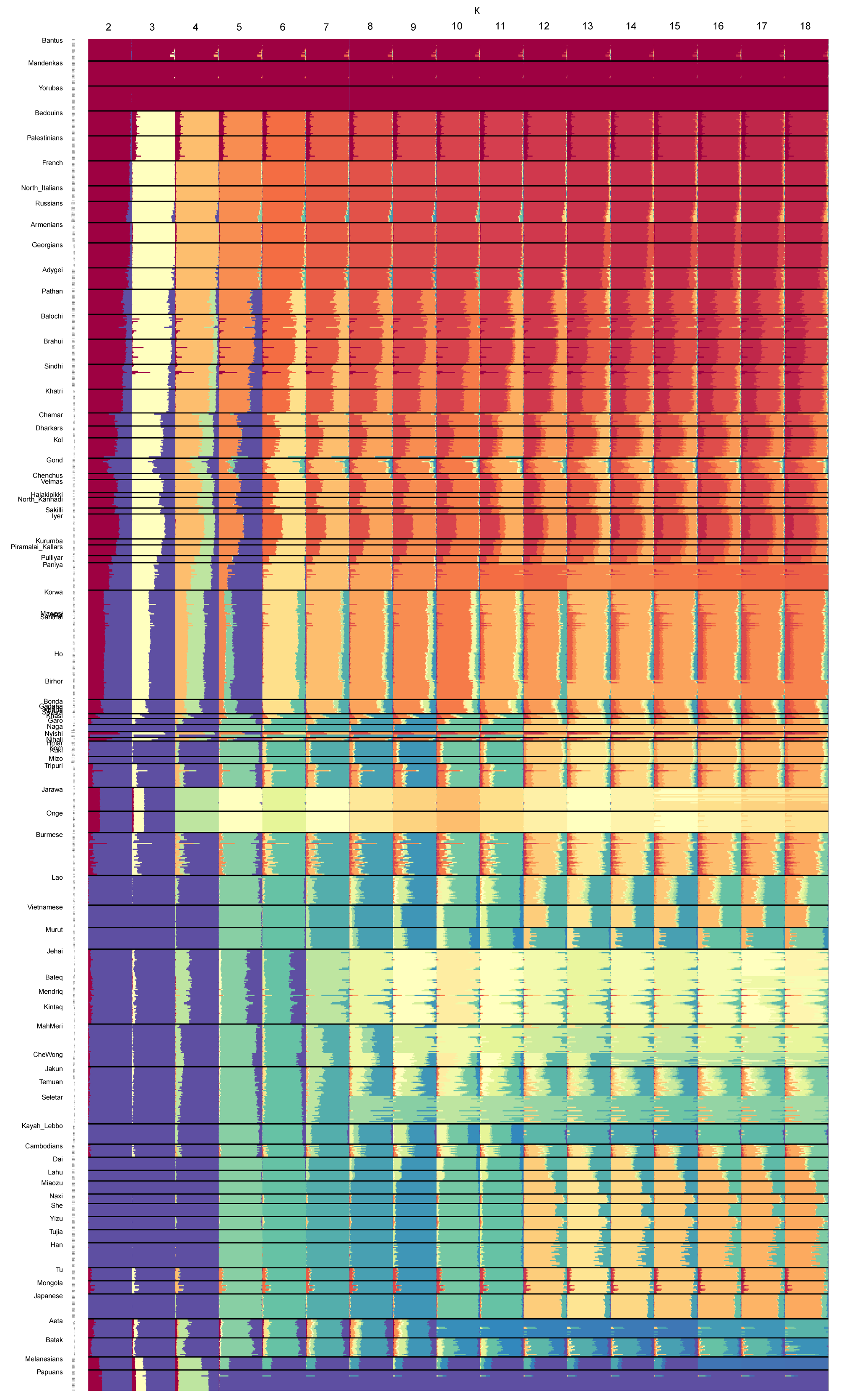
**Supplementary Figure S1.** Results of ADMIXTURE analysis at K=2 to K=18 ancestral components for all the 1072 individuals that remained in the dataset after filtering (Supplementary Table S1). The data was pruned for linkage disequilibrium (LD) using PLINK to exclude SNPs with pairwise genotypic correlation r^2^ > 0.4 in a window of 200 SNPs sliding the window by 25 SNPs at a time. This left us 155743 SNPs on which we ran the ADMIXTURE 1.23 program^1^.


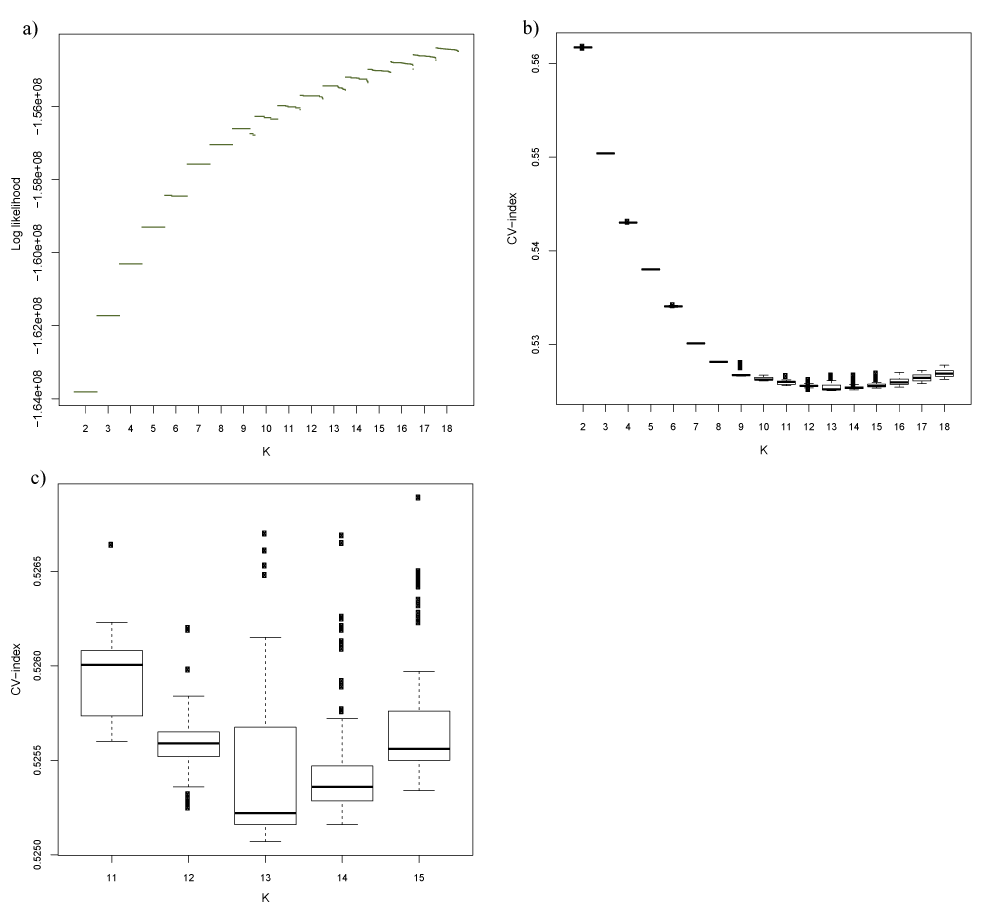


**Supplementary Figure S2.** a) Log likelihood (LL) scores gotten by running ADMIXTURE analysis 100 times for each K. The highest K with stable (global maximum has been reached) LL values is K=13.

b) Based on cross-validation (CV) procedure, genetic structure of a sample set is best described choosing the value of K with the lowest CV error. In our dataset the lowest CV error was at K=13.

c) Zoom in of the CV values at K=11 to K=15. The lowest CV error was at K=13.

**
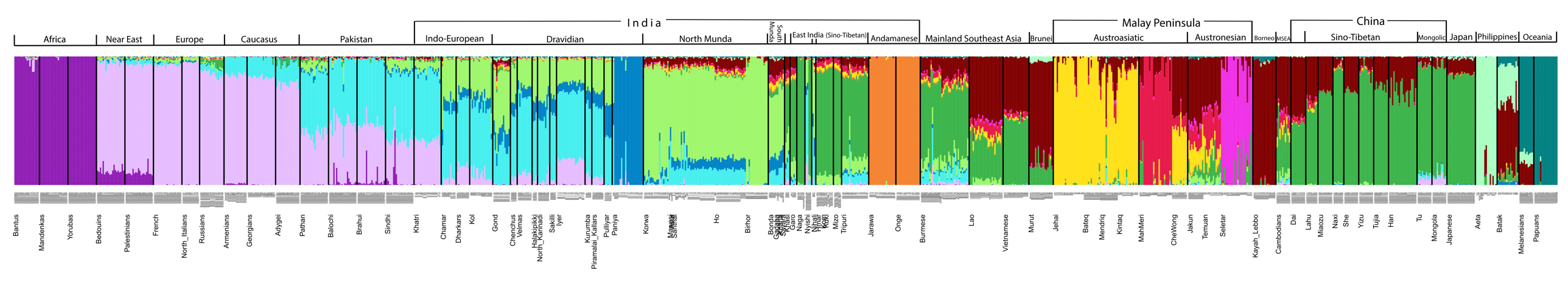
**

**Supplementary Figure S3.** Results of ADMIXTURE analysis at 13 ancestral components. In the upper part, geographical areas and relevant language families are shown. In the lower part, sample IDs and population names are listed.


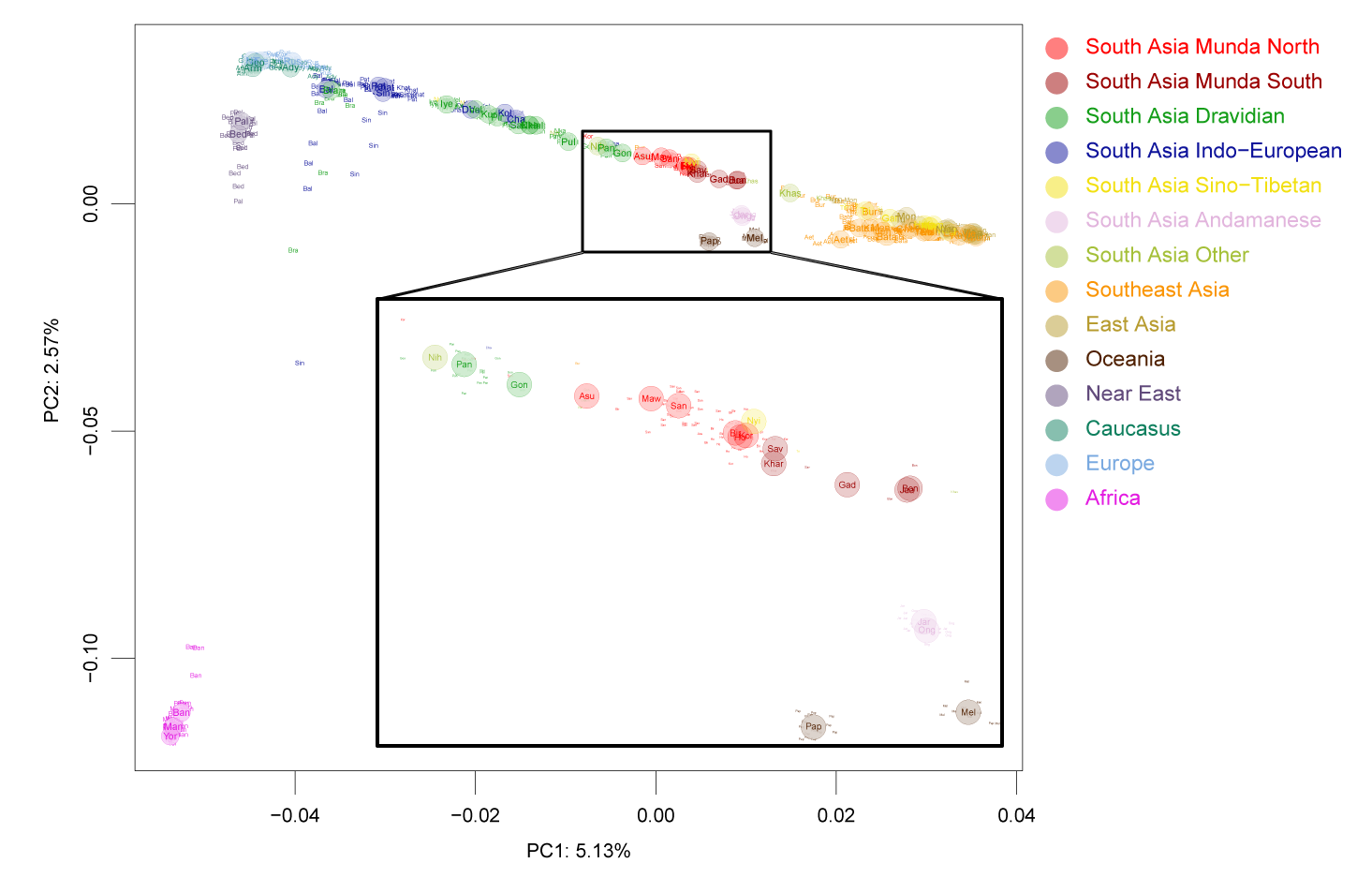


**Supplementary Figure S4.** First two components of principal component analysis (PCA). Individuals and population medians (circles) are marked with abbreviations from population names. Different colours represent populations from different geographic areas and/or linguistic groups as shown on the legend on the right. For the full names of populations see Supplementary Table S1.

PCA was performed using software EIGENSOFT 6.1.4^2^ on the whole filtered dataset (1072 individuals), previously LD pruned as described in the title of Supplementary Figure S1. The first two principal components describe 5.13% and 2.57% of total variance.


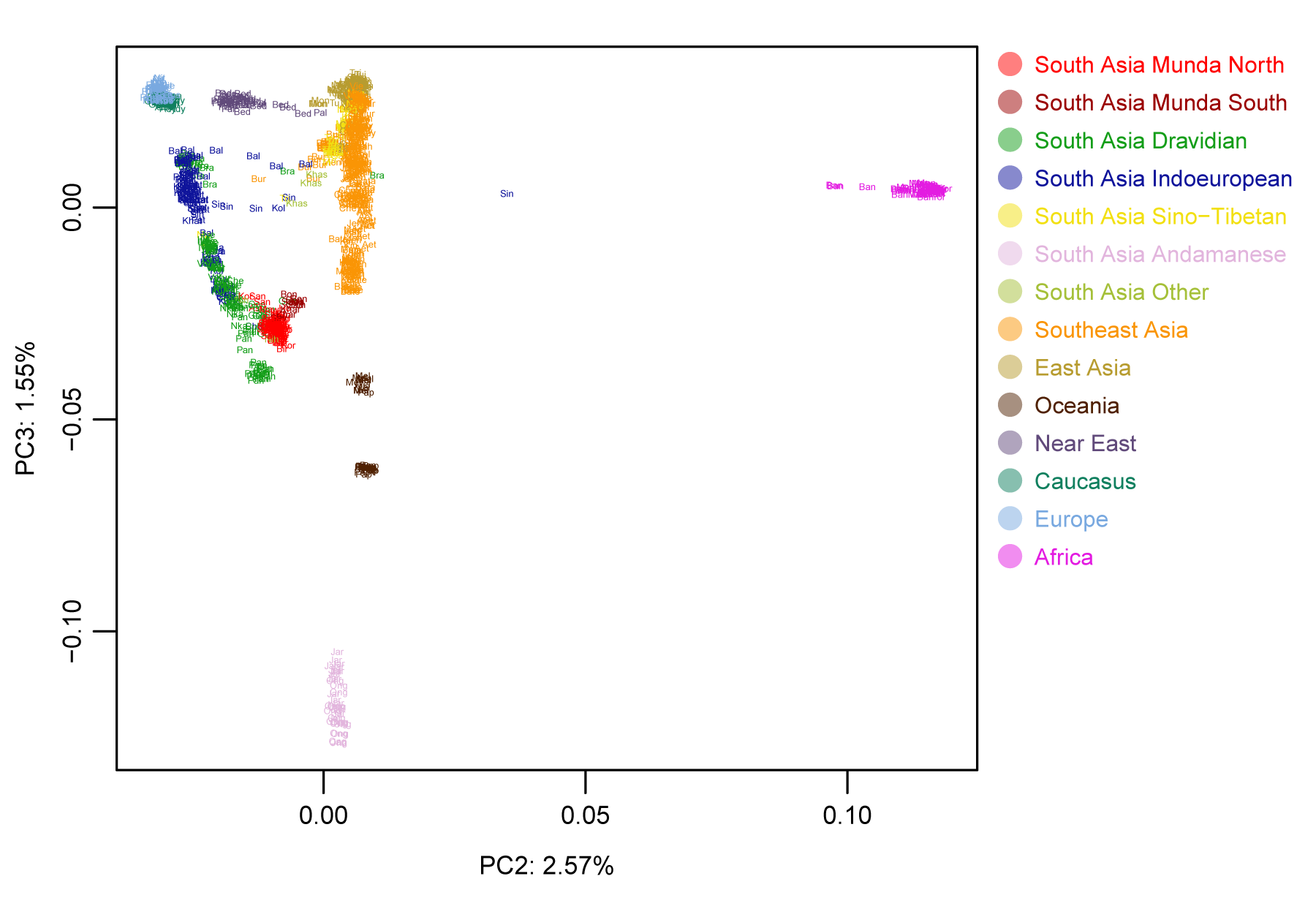


**Supplementary Figure S5.** The second and third component of the global PCA for better visualization of the Munda speakers’ position on Indian ANI-ASI cline. Individuals are marked with abbreviations from population names. Different colours represent populations from different geographic areas and/or linguistic groups as shown on the legend on the right. For the full names of populations see Supplementary Table S1.

PCA was performed using software EIGENSOFT 6.1.4^2^ on the whole filtered dataset (1072 individuals), previously LD pruned as described in the title of Supplementary Figure S1. The second and third principal components describe 2.57% and 1.55% of total variance.

a)

b)

**Supplementary Figure S6.** Chunk-count shared between Andamanese (a) Jarava, b) Onge) and reference groups retrieved using fineSTRUCTURE^3^. For this analysis, the data was previously phased with Beagle 3.3.2^4^. A co-ancestry matrix was constructed using ChromoPainter v1^3^ with the default settings. From the co-ancestry matrix, the mean chunk lengths shared between Eurasian populations and Jarawa and Onge were extracted and plotted with 95% confidence intervals. IE – Indo-European, Dra – Dravidian, NM – North Munda, SM – South Munda, TB – Tibeto-Burman, SEAsia – Southeast Asia, PNG – Papua New Guinea.


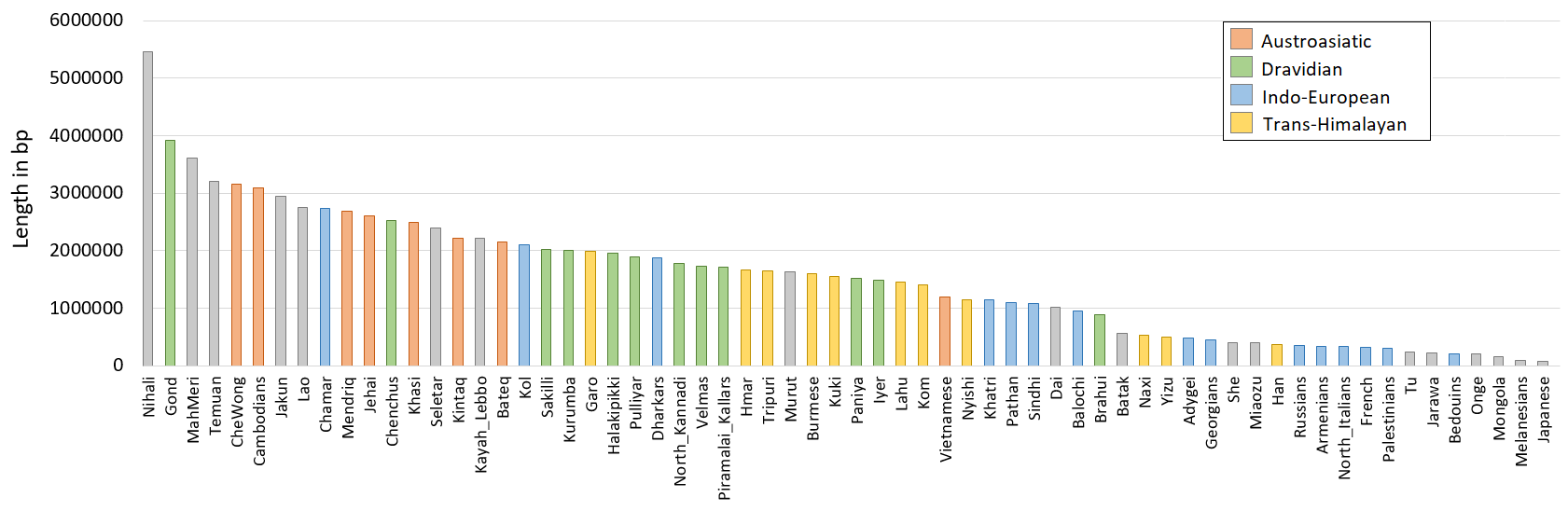


**Supplementary Figure S7.** Total length of DNA segments of identity-by-decent (IBD) shared between a Munda speaker and an individual from a population on x-axis on average, obtained from Refined IBD analysis^5^. The bars are coloured by the four main language families present in India; bars representing populations speaking languages from other language families are grey.


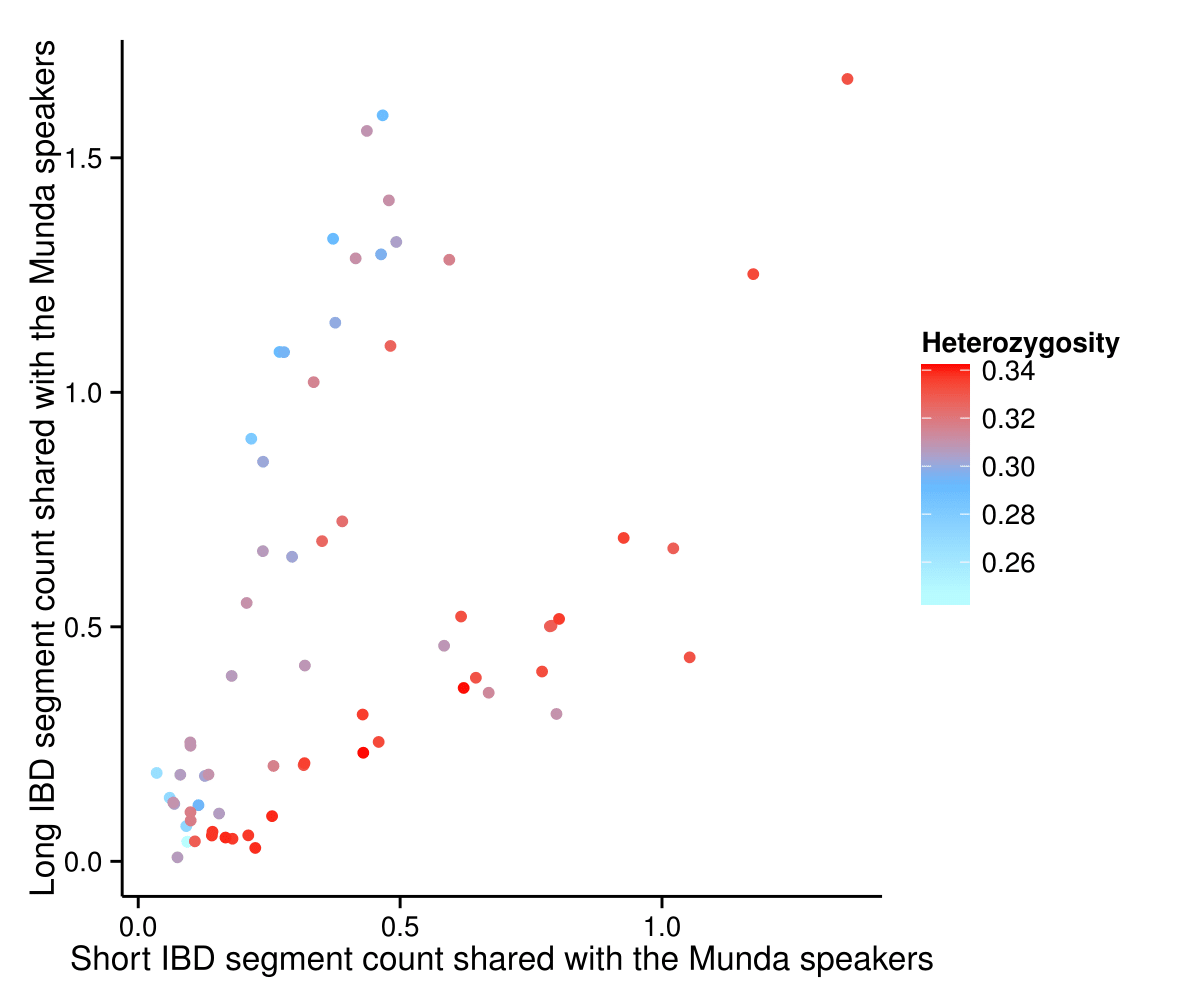


**Supplementary Figure S8.** Average heterozygosity of the populations shown on Figure 2. Populations with lower average heterozygosity (blue) fall in the yellow circle on Figure 2 and populations with higher average heterozygosity (red) fall in the green circle on Figure 2.


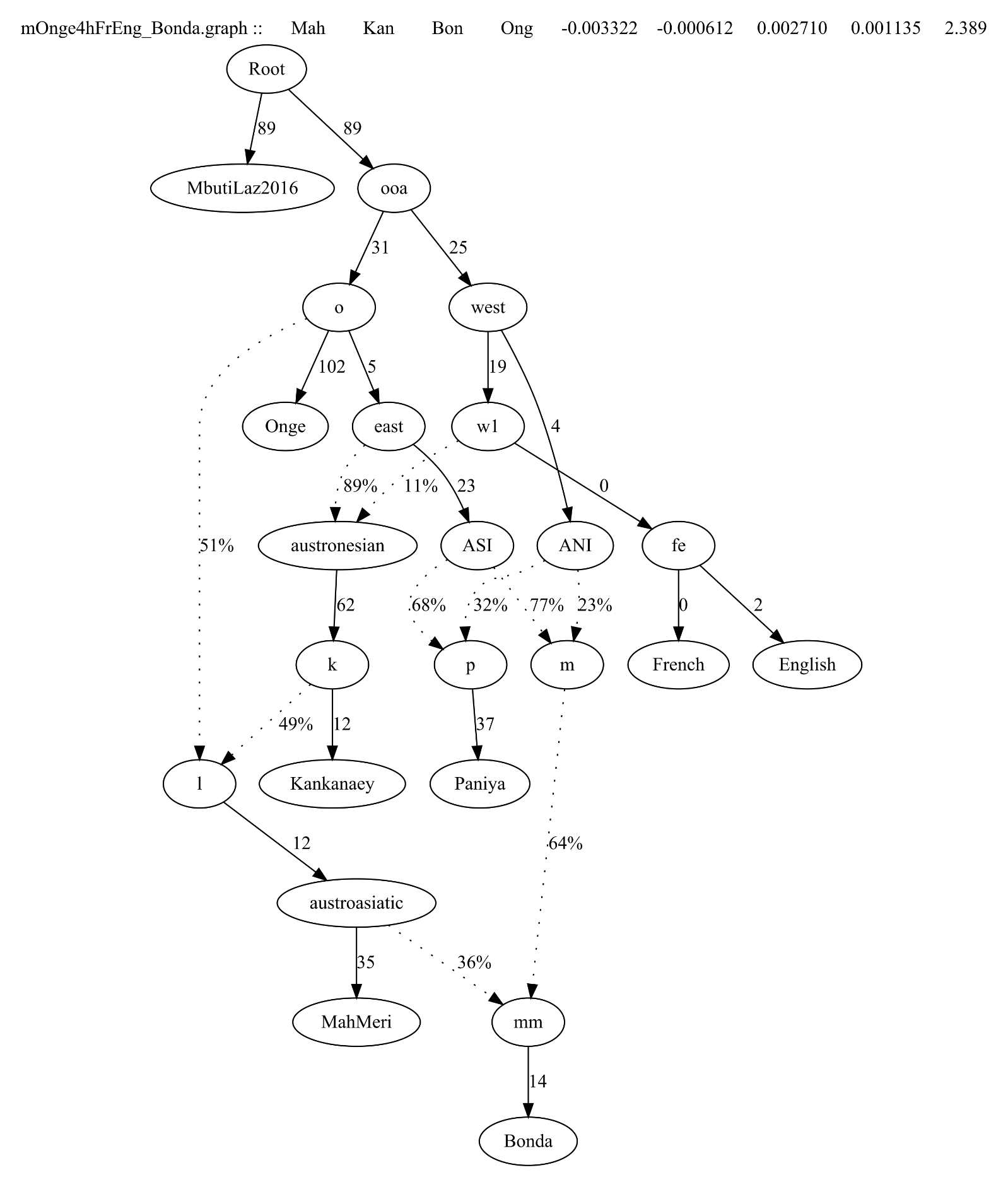


**Supplementary Figure S9.** An eight population qpGraph^6^ model with three distinct ancestry sources for Munda speaking populations. Admixture proportions obtained using this model for 11 Munda populations are reported in Supplementary Table S6.

a)


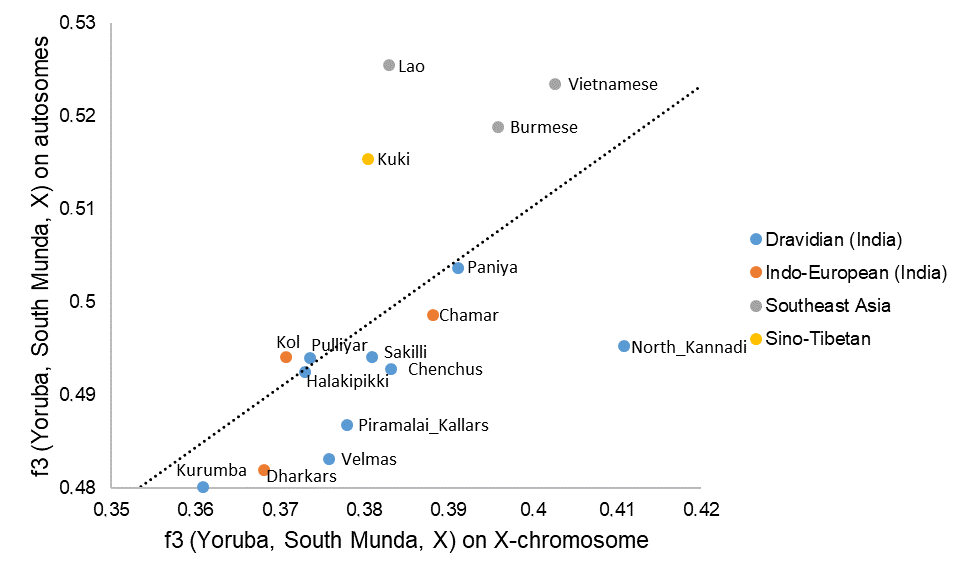


b)


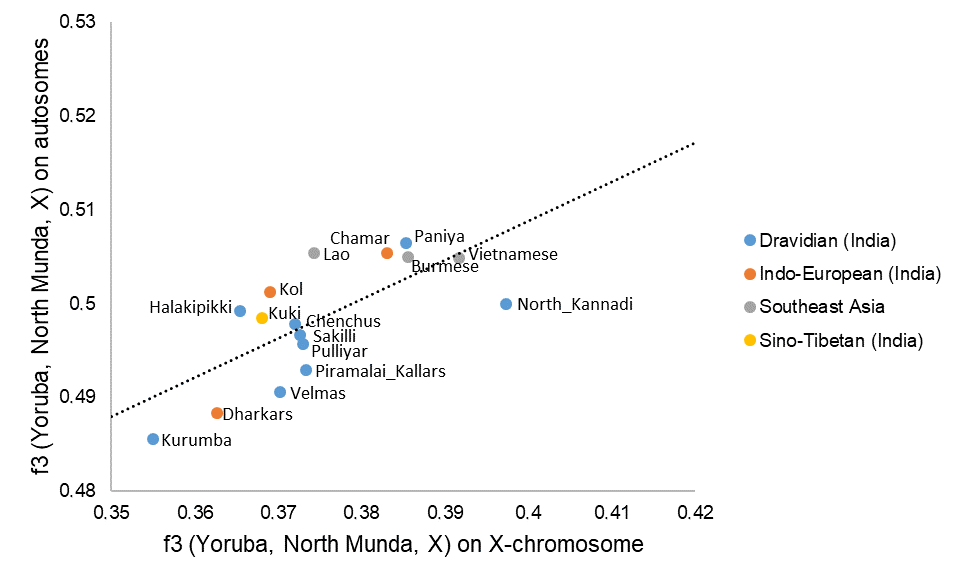


**Supplementary Figure S10.** a) South and b) North Munda average outgroup f3^6^ statistics based on autosomes and X chromosome compared. The dotted line is linear regression line to better visualize which populations are situated on extreme values on either axis.

**
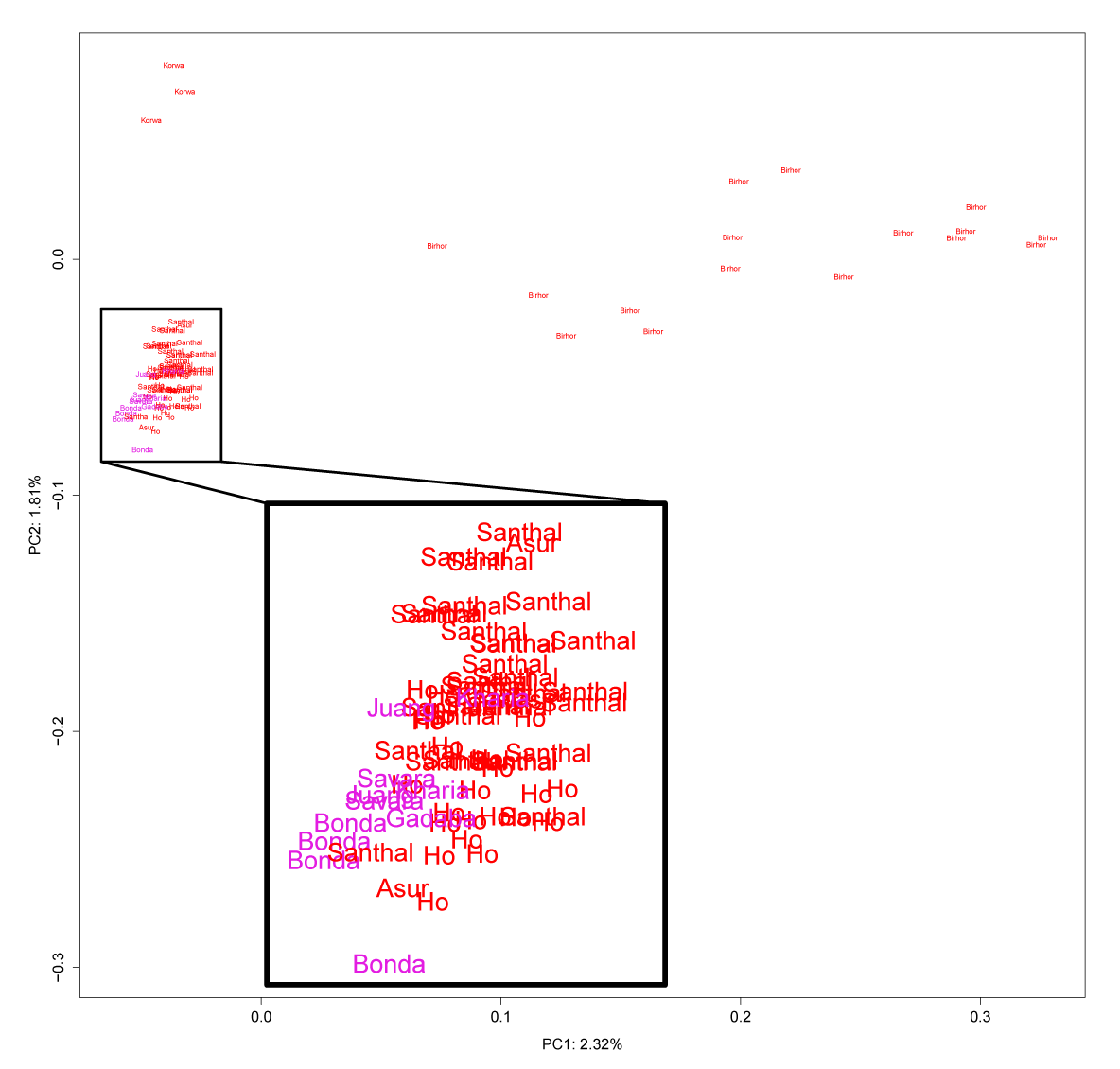
**

**Supplementary Figure S11.** First two components of PCA of only Munda speakers. From South Munda speakers (fuchsia) one Kharia out of two and one Juang out of two fall in the middle of North Munda speakers (red).

**
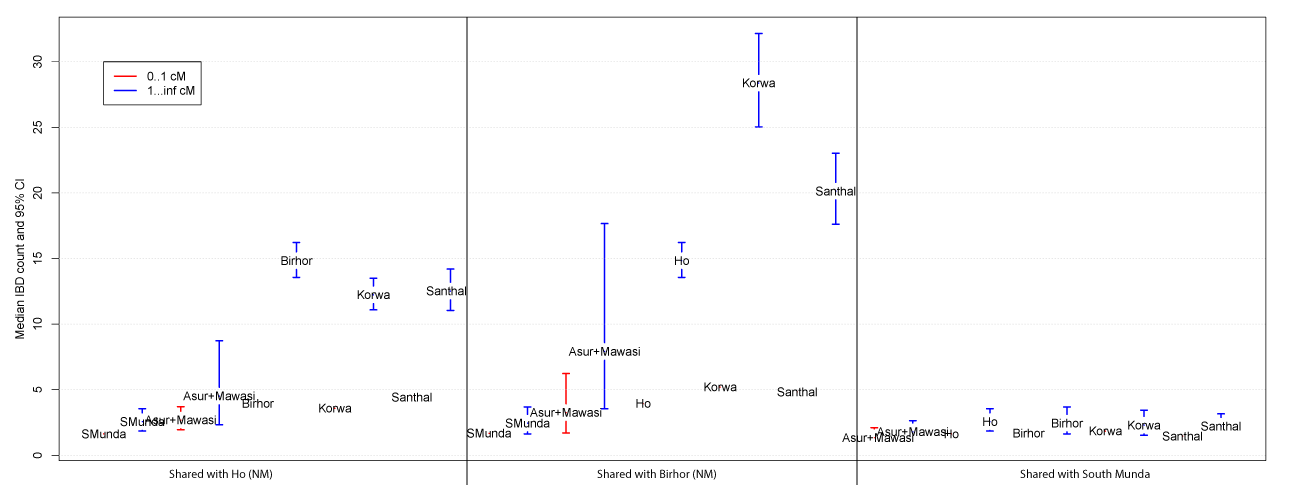
**

**Supplementary Figure S12.** Median IBD segment count between two individuals from different Munda speaking populations. The first third of the graph describes Ho (North Munda) IBD segment sharing with South Mundas and other North Munda populations; the second third of the graph describes Birhor (North Munda) IBD segment sharing with South Mundas and other North Munda populations; the last third describes South Munda IBD segment sharing with different North Munda populations. The shared segments are divided into short (<1 cM) and long (>1 cM). Data points with no visible confidence intervals have actually very narrow intervals. South Munda speakers are treated as one population due to small sample size. Also, North Munda speaking Asur and Mawasi are treated as one population for the same reason.

**
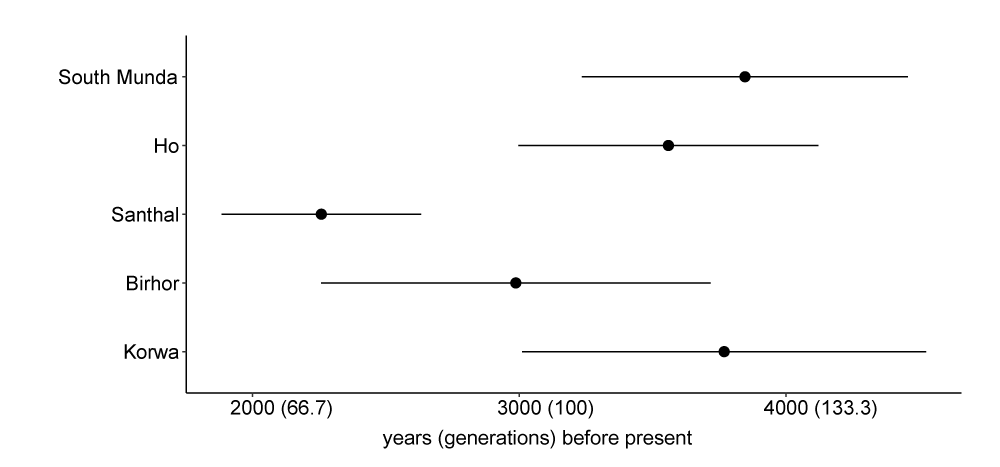
**

**Supplementary Figure S13.** Admixture times as evaluated by ALDER. The population pairs to represent admixture times were chosen based on decay status and LD decay curve amplitude. North Munda speaking Santhal, Ho, Korwa and Birhor were addressed separately as admixed populations; due to a small sample size South Munda speakers were treated as one population. Standard errors are estimated by jackknifing on chromosomes. Generation length is 30 years^7^. For all the pairs, see Supplementary Table S7.

**Supplementary Table S1.** Information on the samples included in any analysis in this paper except qpAdm and qpGraph. [See Excel spreadsheet.]

**Supplementary Table S2.** Results of f3 analysis. For admixture f3, source 1 is a South Asian or West Eurasian population and source 2 is Southeast Asian or East Asian population. Only significant outcomes (|Z|>3) are shown. This table has f3 analysis results for South Munda. All the South Munda speaking tribes (Bonda, Gadaba, Juang, Kharia, Savara) are treated as one population due to small sample size. For North Munda speaking populations as admixture target, see the following sheets of this document. For outgroup f3, see the last two sheets of this document. [See Excel spreadsheet.]

**Supplementary Table S3.** Refined IBD results^5^. Average length, median length, average number of short (<1 cM), average number of long (>1 cM) and average number of all IBD tracts shared between Munda speakers and other populations as detected by Refined IBD analysis. Here, the all the Munda speaking populations are treated as one population. Also, average heterozygosity of each population is displayed.

| Population | Average hetero-zygosity | Average length of IBD segments shared with Munda speakers (bp) | Median length of IBD segments shared with Munda speakers (bp) | Average short IBD count shared with Munda speakers | Average long IBD count shared with Munda speakers | Average IBD count shared with Munda speakers | |
| --- | --- | --- | --- | --- | --- | --- | --- |
| *Piramalai Kallars* | 0.32438 | 1205704 | 961781 | 0.8431854 | 0.51552402 | 1.35870942 |  |
| *Paniya* | 0.31124 | 1286343 | 997766 | 0.66055325 | 0.44810678 | 1.10866003 |  |
| *Iyer* | 0.33978 | 1308530 | 1012222 | 0.69855497 | 0.39499993 | 1.0935549 |  |
| *Velmas* | 0.33031 | 1310833 | 1028985 | 0.81050092 | 0.46507532 | 1.27557624 |  |
| *Pathan* | 0.33103 | 1310942 | 1007605 | 0.5233458 | 0.26329502 | 0.78664082 |  |
| *Sindhi* | 0.33364 | 1316658 | 1029250 | 0.4783092 | 0.2926056 | 0.7709148 |  |
| *North Kannadi* | 0.32581 | 1342409 | 1043733 | 0.8229729 | 0.45775247 | 1.28072537 |  |
| *Halakipikki* | 0.32891 | 1365434 | 953103 | 0.99001106 | 0.42106021 | 1.41107127 |  |
| *Dharkars* | 0.32961 | 1376419 | 981193 | 0.71073029 | 0.58912 | 1.29985029 |  |
| *Lahu* | 0.29976 | 1390682 | 1169182 | 0.30936897 | 0.6924967 | 1.00186567 |  |
| *Sakilli* | 0.32880 | 1395210 | 979066 | 0.87608202 | 0.51654455 | 1.39262657 |  |
| *Brahui* | 0.33321 | 1399958 | 1019709 | 0.36549764 | 0.20933053 | 0.57482817 |  |
| *Balochi* | 0.32844 | 1400155 | 983279 | 0.38951912 | 0.23819162 | 0.62771074 |  |
| *Kol* | 0.33413 | 1404063 | 1076997 | 0.87401459 | 0.57476754 | 1.44878213 |  |
| *Khatri* | 0.34038 | 1410126 | 1028542 | 0.47757128 | 0.28693848 | 0.76450976 |  |
| *Chenchus* | 0.32538 | 1433707 | 1080002 | 1.03305243 | 0.64226412 | 1.67531655 |  |
| *Kurumba* | 0.30798 | 1437939 | 981443 | 0.88527132 | 0.40921124 | 1.29448256 |  |
| *Nyishi* | 0.31435 | 1446835 | 1204169 | 0.28603395 | 0.19340506 | 0.47943901 |  |
| *Chamar* | 0.33282 | 1453558 | 1103581 | 0.9535325 | 0.67221755 | 1.62575005 |  |
| *Pulliyar* | 0.30593 | 1467787 | 1060120 | 0.70737461 | 0.43027227 | 1.13764688 |  |
| *Tripuri* | 0.32064 | 1482547 | 1234669 | 0.41092581 | 0.6795179 | 1.09044371 |  |
| *Jakun* | 0.30679 | 1485902 | 1270616 | 0.44620322 | 1.38963566 | 1.83583888 |  |
| *Murut* | 0.29903 | 1512318 | 1215464 | 0.243595 | 0.77662789 | 1.02022289 |  |
| *CheWong* | 0.29469 | 1514213 | 1253713 | 0.48044332 | 1.42736887 | 1.90781219 |  |
| *Burmese* | 0.32304 | 1533029 | 1225830 | 0.38100266 | 0.61015093 | 0.99115359 |  |
| *Vietnamese* | 0.30832 | 1556256 | 1275759 | 0.21947782 | 0.5240159 | 0.74349372 |  |
| *Mendriq* | 0.30183 | 1556944 | 1211769 | 0.38467755 | 1.1789833 | 1.56366085 |  |
| *Temuan* | 0.30895 | 1565760 | 1256627 | 0.47329799 | 1.45858759 | 1.93188558 |  |
| *Mizo* | 0.30603 | 1585031 | 1217197 | 0.30307771 | 0.57522193 | 0.87829964 |  |
| *Kayah Lebbo* | 0.29352 | 1587542 | 1302343 | 0.31965224 | 0.94504198 | 1.26469422 |  |
| *MahMeri* | 0.28806 | 1589998 | 1251841 | 0.45745657 | 1.58719658 | 2.04465315 |  |
| *Kintaq* | 0.29727 | 1590667 | 1292763 | 0.28512682 | 0.98146663 | 1.26659345 |  |
| *Lao* | 0.30897 | 1620139 | 1289940 | 0.40522733 | 1.19457827 | 1.5998056 |  |
| *Khasi* | 0.32429 | 1622290 | 1253969 | 0.48532104 | 0.98333277 | 1.46865381 |  |
| *Kuki* | 0.30451 | 1627290 | 1258110 | 0.30319171 | 0.62727486 | 0.93046657 |  |
| *Garo* | 0.31344 | 1628744 | 1319050 | 0.35339176 | 0.82869517 | 1.18208693 |  |
| *Seletar* | 0.27856 | 1647562 | 1283195 | 0.24750491 | 0.96884778 | 1.21635269 |  |
| *Jehai* | 0.28939 | 1651282 | 1251475 | 0.32181813 | 1.1346814 | 1.45649953 |  |
| *Bateq* | 0.28809 | 1654692 | 1201109 | 0.26797598 | 0.91310955 | 1.18108553 |  |
| *Dai* | 0.30447 | 1669923 | 1257712 | 0.19195201 | 0.41151125 | 0.60346326 |  |
| *Cambodians* | 0.31419 | 1761915 | 1340467 | 0.46926313 | 1.18349585 | 1.65275898 |  |
| *Hmar+Kom* | 0.30954 | 1812539 | 1324701 | 0.22326775 | 0.59561287 | 0.81888062 |  |
| *Mandenkas* | 0.30972 | 585421 | 585421 | N/A | N/A | N/A |  |
| *Yorubas* | 0.30902 | 618499 | 676452 | N/A | N/A | N/A |  |
| *Bantus* | 0.31098 | 727882 | 754283 | N/A | N/A | N/A |  |
| *Japanese* | 0.30423 | 1086653 | 1072658 | 0.06431569 | 0.02777072 | 0.09208641 |  |
| *Papuans* | 0.24322 | 1197361 | 998933 | 0.07653214 | 0.0136409 | 0.09017304 |  |
| *Mongola* | 0.31512 | 1198030 | 918115 | 0.12050406 | 0.05385748 | 0.17436154 |  |
| *Aeta* | 0.29305 | 1241767 | 1065417 | 0.07844757 | 0.07934917 | 0.15779674 |  |
| *Bedouins* | 0.32613 | 1242870 | 953880 | 0.12026383 | 0.03427706 | 0.15454089 |  |
| *French* | 0.33542 | 1253769 | 937930 | 0.15862278 | 0.06404582 | 0.2226686 |  |
| *Russians* | 0.33433 | 1255321 | 981579 | 0.18256455 | 0.05845218 | 0.24101673 |  |
| *Onge* | 0.26781 | 1259344 | 1205584 | 0.04546396 | 0.11647203 | 0.16193599 |  |
| *Jarawa* | 0.26435 | 1267308 | 1077040 | 0.03848648 | 0.10911506 | 0.14760154 |  |
| *Armenians* | 0.33742 | 1277550 | 1059523 | 0.18625967 | 0.07015466 | 0.25641433 |  |
| *Naga* | 0.30030 | 1278067 | 1103025 | 0.1103352 | 0.15185185 | 0.26218705 |  |
| *Palestinians* | 0.33533 | 1289800 | 1052656 | 0.14280076 | 0.06838779 | 0.21118855 |  |
| *Miaozu* | 0.30373 | 1291205 | 1135968 | 0.13216278 | 0.19464814 | 0.32681092 |  |
| *Tujia* | 0.30361 | 1306732 | 1161697 | 0.12257058 | 0.10849313 | 0.23106371 |  |
| *North Italians* | 0.33461 | 1353795 | 987925 | 0.18567652 | 0.0825196 | 0.26819612 |  |
| *Adygei* | 0.33752 | 1356969 | 1059456 | 0.23031686 | 0.0894362 | 0.31975306 |  |
| *Georgians* | 0.33677 | 1376255 | 1030300 | 0.20972921 | 0.07893129 | 0.2886605 |  |
| *Tu* | 0.31539 | 1405238 | 973721 | 0.10987794 | 0.08343905 | 0.19331699 |  |
| *Yizu* | 0.30721 | 1479741 | 1230191 | 0.09566382 | 0.22849666 | 0.32416048 |  |
| *Gond* | 0.33115 | 1517949 | 1157837 | 1.18376582 | 1.31560061 | 2.49936643 |  |
| *Melanesians* | 0.26923 | 1616559 | 1106733 | 0.09140108 | 0.05413412 | 0.1455352 |  |
| *Nihali* | 0.32927 | 1626777 | 1232482 | 1.31939422 | 1.76063398 | 3.0800282 |  |
| *Batak* | 0.30707 | 1665062 | 1158531 | 0.09540116 | 0.19190855 | 0.28730971 |  |
| *Han* | 0.30752 | 1701126 | 1117310 | 0.08913274 | 0.09600951 | 0.18514225 |  |
| *Naxi* | 0.30787 | 1754205 | 1199540 | 0.11316477 | 0.2035664 | 0.31673117 |  |
| *She* | 0.30247 | 1780833 | 1406331 | 0.09111111 | 0.15159327 | 0.24270438 |  |
| *Asur* | 0.32449 |  |  |  |  |  |  |
| *Mawasi* | 0.32287 |  |  |  |  |  |  |
| *Santhal* | 0.32707 |  |  |  |  |  |  |
| *Ho* | 0.32725 |  |  |  |  |  |  |
| *Korwa* | 0.31639 |  |  |  |  |  |  |
| *Birhor* | 0.31501 |  |  |  |  |  |  |
| *Juang* | 0.31896 |  |  |  |  |  |  |
| *Kharia* | 0.31756 |  |  |  |  |  |  |
| *Bonda* | 0.32073 |  |  |  |  |  |  |
| *Gadaba* | 0.32424 |  |  |  |  |  |  |
| *Savara* | 0.32158 |  |  |  |  |  |  |

**Supplementary Table S4.** Results of qpAdm analysis^6^. We run the qpAdm software testing the following South and North Munda populations (Bonda, Gadaba, Juang, Kharia, Savara, Asur, Birhor, Ho, Korwa, Mawasi, Santhal) as a three ways mixture of all possible combinations of West (Anatolia_N, Armenia_MLBA, Germans, Iran_N, IranianLaz2016), East (Lao, MahMeri, Burmese, Cambodian) and South (Onge, Paniya) Asian groups and using as outgroups the following groups (Natufian, WHG, Han, Kankanaey, Karitiana, MbutiLaz2016, Papuan, Ust_Ishim, Yorubas)^8,9^. We report here all combinations that yielded a qpWave p-value >0.05 and provide average information for the two major sets of admixture, namely: West+East+Paniya (a Dravidian speaking Indian population) and West+East+Onge (an indigenous group from Andaman Islands). Notably, when Paniya are used as South Asian group, the West Asian component is not needed to account for the observed allele frequencies in Munda, while West Asians are need when Onge is used as a proxy for the South Asian substrate in Mundas. Note that Paniya can be modelled as 27% West Asia and 73% Onge. The higher amount of East Asian component in South Munda, compared to North Munda, West Asian component being roughly the same is also noteworthy.

Runs with highest p-value with Paniya and with Onge as South Asian source.

| Average | West Asia | SEA | Paniya |  | West Asia | SEA | Onge |
| --- | --- | --- | --- | --- | --- | --- | --- |
| *Bonda (SM)* | 0.00 | 0.39 | 0.61 | or | 0.13 | 0.36 | 0.51 |
| *Gadaba (SM)* | NA | NA | NA | or | 0.14 | 0.20 | 0.66 |
| *Juang (SM)* | 0.01 | 0.41 | 0.58 | or | NA | NA | NA |
| *Kharia (SM)* | 0.01 | 0.34 | 0.65 | or | 0.17 | 0.31 | 0.52 |
| *Savara (SM)* | 0.00 | 0.26 | 0.74 | or | 0.15 | 0.21 | 0.64 |
| *Average SM* | 0.00 | 0.35 | 0.65 | or | 0.15 | 0.27 | 0.58 |
| *Asur (NM)* | 0.00 | 0.23 | 0.77 | or | 0.14 | 0.17 | 0.69 |
| *Birhor (NM)* | 0.00 | 0.21 | 0.79 | or | 0.14 | 0.20 | 0.66 |
| *Ho (NM)* | 0.00 | 0.25 | 0.75 | or | 0.16 | 0.20 | 0.64 |
| *Korwa (NM)* | 0.00 | 0.25 | 0.75 | or | NA | NA | NA |
| *Mawasi (NM)* | 0.00 | 0.20 | 0.80 | or | 0.18 | 0.17 | 0.65 |
| *Santhal (NM)* | 0.00 | 0.21 | 0.79 | or | NA | NA | NA |
| *Average NM* | 0.00 | 0.23 | 0.78 | or | 0.16 | 0.19 | 0.66 |
| *Total Average* | 0.00 | 0.28 | 0.71 | or | 0.15 | 0.23 | 0.62 |

West Asian component retrieved from Onge run, Southeast Asian component from Paniya run and South Asian component calculated as South Asia = 1 – West Asia – Southeast Asia.

| Average | West Asia | Southeast Asia | South Asia |
| --- | --- | --- | --- |
| *South Munda* | 0.15 | 0.35 | 0.50 |
| *SM_SEA=0* | 0.23 | 0.00 | 0.77 |
| *North Munda* | 0.16 | 0.23 | 0.61 |
| *NM_SEA=0* | 0.21 | 0.00 | 0.79 |
| *Total Average* | 0.155 | 0.29 | 0.555 |
| *Total_Average_SEA=0* | 0.22 | 0.00 | 0.78 |

Raw results with p-value higher than 0.05.

| Recipient | p-value | S1 | S2 | S3 | p1 | p2 | p3 |
| --- | --- | --- | --- | --- | --- | --- | --- |
| *Asur* | 0.078692 | *IranianLaz2016* | *Burmese* | *Onge* | 0.131 | 0.154 | 0.716 |
| *Asur* | 0.077707 | *IranianLaz2016* | *Cambodian* | *Onge* | 0.132 | 0.141 | 0.727 |
| *Asur* | 0.090879 | *IranianLaz2016* | *MahMeri* | *Onge* | 0.142 | 0.166 | 0.693 |
| *Asur* | 0.527424 | *IranianLaz2016* | *Burmese* | *Paniya* | 0 | 0.225 | 0.775 |
| *Asur* | 0.540888 | *IranianLaz2016* | *Cambodian* | *Paniya* | 0 | 0.21 | 0.79 |
| *Asur* | 0.683904 | *IranianLaz2016* | *MahMeri* | *Paniya* | 0 | 0.23 | 0.77 |
| *Birhor* | 0.231668 | *IranianLaz2016* | *Burmese* | *Onge* | 0.131 | 0.189 | 0.68 |
| *Birhor* | 0.152635 | *IranianLaz2016* | *Cambodian* | *Onge* | 0.134 | 0.171 | 0.695 |
| *Birhor* | 0.127584 | *IranianLaz2016* | *MahMeri* | *Onge* | 0.142 | 0.195 | 0.663 |
| *Birhor* | 0.058091 | *IranianLaz2016* | *Burmese* | *Paniya* | 0 | 0.257 | 0.743 |
| *Bonda* | 0.059023 | *IranianLaz2016* | *Burmese* | *Onge* | 0.1 | 0.32 | 0.58 |
| *Bonda* | 0.413967 | *IranianLaz2016* | *Cambodian* | *Onge* | 0.115 | 0.311 | 0.575 |
| *Bonda* | 0.56112 | *IranianLaz2016* | *MahMeri* | *Onge* | 0.133 | 0.357 | 0.51 |
| *Bonda* | 0.219843 | *IranianLaz2016* | *Cambodian* | *Paniya* | 0 | 0.359 | 0.641 |
| *Bonda* | 0.456687 | *IranianLaz2016* | *MahMeri* | *Paniya* | 0 | 0.392 | 0.608 |
| *Gadaba* | 0.112651 | *IranianLaz2016* | *Burmese* | *Onge* | 0.125 | 0.247 | 0.628 |
| *Gadaba* | 0.078937 | *IranianLaz2016* | *Cambodian* | *Onge* | 0.129 | 0.226 | 0.645 |
| *Ho* | 0.104516 | *IranianLaz2016* | *Burmese* | *Onge* | 0.139 | 0.176 | 0.685 |
| *Ho* | 0.256285 | *IranianLaz2016* | *Cambodian* | *Onge* | 0.146 | 0.17 | 0.684 |
| *Ho* | 0.397776 | *IranianLaz2016* | *MahMeri* | *Onge* | 0.157 | 0.199 | 0.644 |
| *Ho* | 0.052115 | *IranianLaz2016* | *Cambodian* | *Paniya* | 0 | 0.231 | 0.769 |
| *Ho* | 0.231915 | *IranianLaz2016* | *MahMeri* | *Paniya* | 0 | 0.251 | 0.749 |
| *Juang* | 0.2003 | *IranianLaz2016* | *Burmese* | *Paniya* | 0 | 0.388 | 0.612 |
| *Juang* | 0.63607 | *IranianLaz2016* | *Cambodian* | *Paniya* | 0 | 0.368 | 0.632 |
| *Juang* | 0.326936 | *IranianLaz2016* | *MahMeri* | *Paniya* | 0.023 | 0.412 | 0.566 |
| *Juang* | 0.407671 | *IranianLaz2016* | *MahMeri* | *Paniya* | 0 | 0.399 | 0.601 |
| *Kharia* | 0.427576 | *IranianLaz2016* | *Burmese* | *Onge* | 0.141 | 0.285 | 0.574 |
| *Kharia* | 0.76246 | *IranianLaz2016* | *Cambodian* | *Onge* | 0.151 | 0.272 | 0.577 |
| *Kharia* | 0.909329 | *IranianLaz2016* | *MahMeri* | *Onge* | 0.167 | 0.315 | 0.518 |
| *Kharia* | 0.498938 | *IranianLaz2016* | *Burmese* | *Paniya* | 0 | 0.327 | 0.673 |
| *Kharia* | 0.961323 | *IranianLaz2016* | *Cambodian* | *Paniya* | 0 | 0.309 | 0.691 |
| *Kharia* | 0.945664 | *IranianLaz2016* | *MahMeri* | *Paniya* | 0.017 | 0.341 | 0.641 |
| *Kharia* | 0.966397 | *IranianLaz2016* | *MahMeri* | *Paniya* | 0 | 0.332 | 0.668 |
| *Korwa* | 0.134996 | *IranianLaz2016* | *Cambodian* | *Paniya* | 0 | 0.23 | 0.77 |
| *Korwa* | 0.334495 | *IranianLaz2016* | *MahMeri* | *Paniya* | 0 | 0.25 | 0.75 |
| *Mawasi* | 0.268657 | *IranianLaz2016* | *Burmese* | *Onge* | 0.176 | 0.156 | 0.668 |
| *Mawasi* | 0.249444 | *IranianLaz2016* | *Cambodian* | *Onge* | 0.177 | 0.144 | 0.679 |
| *Mawasi* | 0.238949 | *IranianLaz2016* | *MahMeri* | *Onge* | 0.185 | 0.166 | 0.649 |
| *Mawasi* | 0.374481 | *IranianLaz2016* | *Burmese* | *Paniya* | 0 | 0.195 | 0.805 |
| *Mawasi* | 0.389036 | *IranianLaz2016* | *Cambodian* | *Paniya* | 0 | 0.182 | 0.818 |
| *Mawasi* | 0.358015 | *IranianLaz2016* | *MahMeri* | *Paniya* | 0 | 0.198 | 0.802 |
| *Santhal* | 0.783093 | *IranianLaz2016* | *Burmese* | *Paniya* | 0 | 0.212 | 0.788 |
| *Santhal* | 0.778974 | *IranianLaz2016* | *Cambodian* | *Paniya* | 0 | 0.196 | 0.804 |
| *Santhal* | 0.519636 | *IranianLaz2016* | *MahMeri* | *Paniya* | 0 | 0.209 | 0.791 |
| *Savara* | 0.386645 | *IranianLaz2016* | *Burmese* | *Onge* | 0.135 | 0.199 | 0.665 |
| *Savara* | 0.535614 | *IranianLaz2016* | *Cambodian* | *Onge* | 0.143 | 0.192 | 0.665 |
| *Savara* | 0.320532 | *IranianLaz2016* | *MahMeri* | *Onge* | 0.15 | 0.21 | 0.64 |
| *Savara* | 0.12299 | *IranianLaz2016* | *Cambodian* | *Paniya* | 0 | 0.255 | 0.745 |
| *Asur* | 0.086974 | *IranianLaz2016* | *Lao* | *Onge* | 0.143 | 0.126 | 0.731 |
| *Birhor* | 0.213122 | *IranianLaz2016* | *Lao* | *Onge* | 0.145 | 0.154 | 0.701 |
| *Bonda* | 0.511882 | *IranianLaz2016* | *Lao* | *Onge* | 0.133 | 0.275 | 0.591 |
| *Gadaba* | 0.086767 | *IranianLaz2016* | *Lao* | *Onge* | 0.141 | 0.201 | 0.658 |
| *Ho* | 0.370207 | *IranianLaz2016* | *Lao* | *Onge* | 0.158 | 0.153 | 0.689 |
| *Kharia* | 0.890318 | *IranianLaz2016* | *Lao* | *Onge* | 0.169 | 0.245 | 0.586 |
| *Mawasi* | 0.287968 | *IranianLaz2016* | *Lao* | *Onge* | 0.188 | 0.132 | 0.681 |
| *Savara* | 0.484997 | *IranianLaz2016* | *Lao* | *Onge* | 0.153 | 0.168 | 0.679 |
| *Asur* | 0.660876 | *Anatolia_N* | *Lao* | *Paniya* | 0 | 0.184 | 0.816 |
| *Asur* | 0.62012 | *Armenia_MLBA* | *Lao* | *Paniya* | 0 | 0.181 | 0.819 |
| *Asur* | 0.660876 | *Germans* | *Lao* | *Paniya* | 0 | 0.184 | 0.816 |
| *Asur* | 0.661196 | *Iran_N* | *Lao* | *Paniya* | 0 | 0.184 | 0.816 |
| *Asur* | 0.660876 | *IranianLaz2016* | *Lao* | *Paniya* | 0 | 0.184 | 0.816 |
| *Birhor* | 0.07334 | *Anatolia_N* | *Lao* | *Paniya* | 0 | 0.209 | 0.791 |
| *Birhor* | 0.07334 | *Germans* | *Lao* | *Paniya* | 0 | 0.209 | 0.791 |
| *Birhor* | 0.073824 | *Iran_N* | *Lao* | *Paniya* | 0 | 0.209 | 0.791 |
| *Birhor* | 0.07334 | *IranianLaz2016* | *Lao* | *Paniya* | 0 | 0.209 | 0.791 |
| *Bonda* | 0.286785 | *Anatolia_N* | *Lao* | *Paniya* | 0 | 0.314 | 0.686 |
| *Bonda* | 0.256872 | *Armenia_MLBA* | *Lao* | *Paniya* | 0 | 0.307 | 0.693 |
| *Bonda* | 0.286785 | *Germans* | *Lao* | *Paniya* | 0 | 0.314 | 0.686 |
| *Bonda* | 0.286715 | *Iran_N* | *Lao* | *Paniya* | 0 | 0.314 | 0.686 |
| *Bonda* | 0.286785 | *IranianLaz2016* | *Lao* | *Paniya* | 0 | 0.314 | 0.686 |
| *Ho* | 0.1214 | *Anatolia_N* | *Lao* | *Paniya* | 0 | 0.202 | 0.798 |
| *Ho* | 0.094341 | *Armenia_MLBA* | *Lao* | *Paniya* | 0 | 0.197 | 0.803 |
| *Ho* | 0.1214 | *Germans* | *Lao* | *Paniya* | 0 | 0.202 | 0.798 |
| *Ho* | 0.119824 | *Iran_N* | *Lao* | *Paniya* | 0 | 0.202 | 0.798 |
| *Ho* | 0.1214 | *IranianLaz2016* | *Lao* | *Paniya* | 0 | 0.202 | 0.798 |
| *Juang* | 0.474513 | *Anatolia_N* | *Lao* | *Paniya* | 0.005 | 0.32 | 0.675 |
| *Juang* | 0.561935 | *Anatolia_N* | *Lao* | *Paniya* | 0 | 0.322 | 0.678 |
| *Juang* | 0.5953 | *Armenia_MLBA* | *Lao* | *Paniya* | 0.004 | 0.32 | 0.676 |
| *Juang* | 0.696344 | *Armenia_MLBA* | *Lao* | *Paniya* | 0 | 0.321 | 0.679 |
| *Juang* | 0.489269 | *Germans* | *Lao* | *Paniya* | 0.009 | 0.32 | 0.671 |
| *Juang* | 0.561935 | *Germans* | *Lao* | *Paniya* | 0 | 0.322 | 0.678 |
| *Juang* | 0.450814 | *Iran_N* | *Lao* | *Paniya* | 0.004 | 0.322 | 0.675 |
| *Juang* | 0.562041 | *Iran_N* | *Lao* | *Paniya* | 0 | 0.322 | 0.678 |
| *Juang* | 0.444731 | *IranianLaz2016* | *Lao* | *Paniya* | 0.001 | 0.323 | 0.676 |
| *Juang* | 0.561935 | *IranianLaz2016* | *Lao* | *Paniya* | 0 | 0.322 | 0.678 |
| *Kharia* | 0.949199 | *Anatolia_N* | *Lao* | *Paniya* | 0.002 | 0.27 | 0.728 |
| *Kharia* | 0.975621 | *Anatolia_N* | *Lao* | *Paniya* | 0 | 0.27 | 0.73 |
| *Kharia* | 0.928393 | *Armenia_MLBA* | *Lao* | *Paniya* | 0.002 | 0.268 | 0.731 |
| *Kharia* | 0.964287 | *Armenia_MLBA* | *Lao* | *Paniya* | 0 | 0.268 | 0.732 |
| *Kharia* | 0.947201 | *Germans* | *Lao* | *Paniya* | 0.001 | 0.27 | 0.729 |
| *Kharia* | 0.975621 | *Germans* | *Lao* | *Paniya* | 0 | 0.27 | 0.73 |
| *Kharia* | 0.946124 | *Iran_N* | *Lao* | *Paniya* | 0.002 | 0.27 | 0.728 |
| *Kharia* | 0.974572 | *Iran_N* | *Lao* | *Paniya* | 0 | 0.27 | 0.73 |
| *Kharia* | 0.975621 | *IranianLaz2016* | *Lao* | *Paniya* | 0 | 0.27 | 0.73 |
| *Korwa* | 0.368878 | *Anatolia_N* | *Lao* | *Paniya* | 0 | 0.202 | 0.798 |
| *Korwa* | 0.22085 | *Armenia_MLBA* | *Lao* | *Paniya* | 0 | 0.194 | 0.806 |
| *Korwa* | 0.368878 | *Germans* | *Lao* | *Paniya* | 0 | 0.202 | 0.798 |
| *Korwa* | 0.368865 | *Iran_N* | *Lao* | *Paniya* | 0 | 0.202 | 0.798 |
| *Korwa* | 0.368878 | *IranianLaz2016* | *Lao* | *Paniya* | 0 | 0.202 | 0.798 |
| *Mawasi* | 0.434982 | *Anatolia_N* | *Lao* | *Paniya* | 0 | 0.161 | 0.839 |
| *Mawasi* | 0.26814 | *Armenia_MLBA* | *Lao* | *Paniya* | 0 | 0.148 | 0.852 |
| *Mawasi* | 0.434982 | *Germans* | *Lao* | *Paniya* | 0 | 0.161 | 0.839 |
| *Mawasi* | 0.32534 | *Iran_N* | *Lao* | *Paniya* | 0.004 | 0.161 | 0.835 |
| *Mawasi* | 0.431084 | *Iran_N* | *Lao* | *Paniya* | 0 | 0.161 | 0.839 |
| *Mawasi* | 0.434982 | *IranianLaz2016* | *Lao* | *Paniya* | 0 | 0.161 | 0.839 |
| *Santhal* | 0.708787 | *Anatolia_N* | *Lao* | *Paniya* | 0 | 0.17 | 0.829 |
| *Santhal* | 0.806144 | *Anatolia_N* | *Lao* | *Paniya* | 0 | 0.17 | 0.83 |
| *Santhal* | 0.607252 | *Armenia_MLBA* | *Lao* | *Paniya* | 0.002 | 0.166 | 0.832 |
| *Santhal* | 0.708526 | *Armenia_MLBA* | *Lao* | *Paniya* | 0 | 0.166 | 0.834 |
| *Santhal* | 0.709011 | *Germans* | *Lao* | *Paniya* | 0.001 | 0.17 | 0.829 |
| *Santhal* | 0.806144 | *Germans* | *Lao* | *Paniya* | 0 | 0.17 | 0.83 |
| *Santhal* | 0.720903 | *Iran_N* | *Lao* | *Paniya* | 0.003 | 0.171 | 0.826 |
| *Santhal* | 0.80653 | *Iran_N* | *Lao* | *Paniya* | 0 | 0.17 | 0.83 |
| *Santhal* | 0.806144 | *IranianLaz2016* | *Lao* | *Paniya* | 0 | 0.17 | 0.83 |
| *Savara* | 0.075226 | *Anatolia_N* | *Lao* | *Paniya* | 0.013 | 0.217 | 0.77 |
| *Savara* | 0.074306 | *Anatolia_N* | *Lao* | *Paniya* | 0 | 0.22 | 0.78 |
| *Savara* | 0.066081 | *Armenia_MLBA* | *Lao* | *Paniya* | 0.017 | 0.216 | 0.767 |
| *Savara* | 0.065565 | *Armenia_MLBA* | *Lao* | *Paniya* | 0 | 0.218 | 0.782 |
| *Savara* | 0.058491 | *Germans* | *Lao* | *Paniya* | 0.013 | 0.218 | 0.768 |
| *Savara* | 0.074306 | *Germans* | *Lao* | *Paniya* | 0 | 0.22 | 0.78 |
| *Savara* | 0.056864 | *Iran_N* | *Lao* | *Paniya* | 0.015 | 0.219 | 0.766 |
| *Savara* | 0.074777 | *Iran_N* | *Lao* | *Paniya* | 0 | 0.22 | 0.78 |
| *Savara* | 0.074306 | *IranianLaz2016* | *Lao* | *Paniya* | 0 | 0.22 | 0.78 |

**Supplementary Table S5.** Outgroup f4^6^ analysis to determine if the Munda speakers could be grouped into meta-populations^8^ for qpGraph analysis. Pairs of all Munda populations are tested (Munda1, Munda2). If |Z| score is less than 3 for all the references of a pair, two Munda populations could be treated as one. [See Excel spreadsheet.]

**Supplementary Table S6.** qpGraph and qpAdm estimates of three distinct ancestry proportions in Indian Munda speaking populations. Populations used in the qpGraph model are MbutiLaz2016 (outgroup), French, English, Onge, Kankaney, Mah Meri, Paniya and 11 Munda populations (tested one at a time). qpAdm proportions are calculated as follows: West Asia is IranianLaz2016 proportion from IranianLaz2016, Mah Meri and Onge run; Southeast Asia is Mah Meri proportion from Iranian, Mah Meri and Paniya run; South Asia = 1 – West Asia – Southeast Asia. There are empty cells in qpAdm table as the program did not yield any results with p-value higher than 0.05 for those runs. Outgroup populations used in case of all qpAdm runs are Natufian, WHG, Han, Kankanaey, Karitiana, MbutiLaz2016, Papuan, Ust_Ishim, Yorubas^8,9^ and populations used to retrieve proportions are IranianLaz2016, Mah Meri, Onge, Paniya, 11 Munda populations. SM - South Munda, NM - North Munda. Highest |Z score| is reported for the worst f4 statistic for each Munda population used in the model of Supplementary Figure S9.

|  | qpGraph | | | | qpAdm | | |
| --- | --- | --- | --- | --- | --- | --- | --- |
| Munda population | **West Asia** | **South Asia** | **Southeast Asia** | **Highest \|Z score\|** | **West Asia** | **South Asia** | **Southeast Asia** |
| *Bonda (SM)* | 0.15 | 0.49 | 0.36 | 2.39 | 0.13 | 0.48 | 0.39 |
| *Juang (SM)* | 0.16 | 0.49 | 0.35 | 2.37 |  |  | 0.41 |
| *Gadaba (SM)* | 0.19 | 0.48 | 0.33 | 2.96 | 0.14 |  |  |
| *Savara (SM)* | 0.22 | 0.46 | 0.32 | 2.33 | 0.15 | 0.60 | 0.26 |
| *Kharia (SM)* | 0.21 | 0.50 | 0.29 | 2.40 | 0.17 | 0.50 | 0.34 |
| *Average SM* | 0.18 | 0.49 | 0.33 | 2.49 | 0.15 | 0.52 | 0.35 |
| *Median SM* | 0.19 | 0.49 | 0.33 | 2.39 | 0.15 | 0.50 | 0.36 |
| *Ho (NM)* | 0.21 | 0.58 | 0.21 | 2.59 | 0.16 | 0.59 | 0.25 |
| *Mawasi (NM)* | 0.30 | 0.55 | 0.15 | 2.46 | 0.19 | 0.62 | 0.20 |
| *Birhor (NM)* | 0.19 | 0.65 | 0.16 | 2.56 | 0.14 |  |  |
| *Korwa (NM)* | 0.20 | 0.54 | 0.26 | 2.50 |  |  | 0.25 |
| *Santhal (NM)* | 0.25 | 0.54 | 0.21 | 2.63 |  |  | 0.21 |
| *Asur (NM)* | 0.24 | 0.55 | 0.21 | 2.39 | 0.14 | 0.63 | 0.23 |
| *Average NM* | 0.23 | 0.57 | 0.20 | 2.52 | 0.16 | 0.61 | 0.23 |
| *Median NM* | 0.23 | 0.55 | 0.21 | 2.53 | 0.15 | 0.62 | 0.23 |
| *Average all* | 0.21 | 0.53 | 0.27 | 2.51 | 0.15 | 0.57 | 0.29 |
| *Median all* | 0.21 | 0.52 | 0.27 | 2.46 | 0.15 | 0.56 | 0.30 |

**Supplementary Table S7.** ALDER^10^ results for Munda speaking populations. Note that there is a separate sheet for each population. Successfully fitted admixture dates are reported in generations before present (GBP) and in years before present (YBP). For conversion, generation length of 30 years is used^10^. For choosing the most accurate pair, decay status and LD decay curve amplitude were used. On Figure 3, only population pairs, where sources are from South Asia and Southeast Asia, as suggested by Refined IBD and outgroup f3 analyses, are used. Supplementary Figure S13, which displays admixture times of the source population pairs which are chosen based on decay status and LD decay curve amplitude, not suggestions from other analyses, shows a similar picture. [See Excel spreadsheet.]

1. Alexander, D. H., Novembre, J. & Lange, K. Fast model-based estimation of ancestry in unrelated individuals. *Genome Res.* **19,** 1655–1664 (2009).

2. Patterson, N., Price, A. L. & Reich, D. Population Structure and Eigenanalysis. *PLOS Genet.* **2,** e190 (2006).

3. Lawson, D. J., Hellenthal, G., Myers, S. & Falush, D. Inference of Population Structure using Dense Haplotype Data. *PLOS Genet.* **8,** e1002453 (2012).

4. Browning, S. R. & Browning, B. L. Rapid and Accurate Haplotype Phasing and Missing-Data Inference for Whole-Genome Association Studies By Use of Localized Haplotype Clustering. *Am. J. Hum. Genet.* **81,** 1084–1097 (2007).

5. Browning, B. L. & Browning, S. R. Improving the Accuracy and Efficiency of Identity-by-Descent Detection in Population Data. *Genetics* **194,** 459–471 (2013).

6. Patterson, N. *et al.* Ancient admixture in human history. *Genetics* **192,** 1065–1093 (2012).

7. Fenner, J. N. Cross-cultural estimation of the human generation interval for use in genetics-based population divergence studies. *Am. J. Phys. Anthropol.* **128,** 415–423 (2005).

8. Lazaridis, I. *et al.* Genomic insights into the origin of farming in the ancient Near East. *Nature* **536,** 419–424 (2016).

9. Haak, W. *et al.* Massive migration from the steppe was a source for Indo-European languages in Europe. *Nature* **522,** 207–211 (2015).

10. Loh, P.-R. *et al.* Inferring Admixture Histories of Human Populations Using Linkage Disequilibrium. *Genetics* **193,** 1233–1254 (2013).
